# Supplementary material for: Outcomes at Patient and Limb Levels in Peripheral Artery Disease by the Location of Atherosclerotic Lower Limb Lesions: An Observational Study from a High-Volume German Center
Source: J Clin Med. 2025 Oct 4;14(19):7037. doi: 10.3390/jcm14197037 (PMC12525090; doi:10.3390/jcm14197037)
Supplement: Supplementary file 1 [file jcm-14-07037-s001.zip › jcm-3864194-supplementary.pdf]

Supplementary Materials

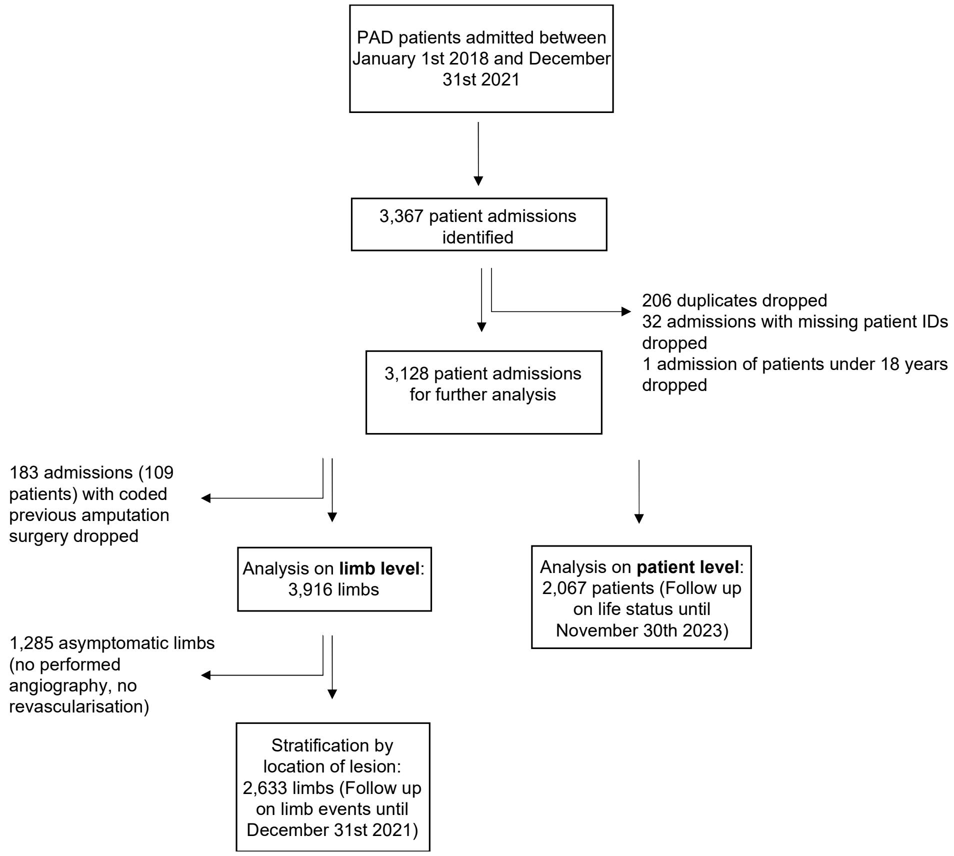

Figure S1: Flowchart of inclusion: Patient cohort. Details in Supp. Table 1.

**ICD Diagnoses**
Main diagnosis: ICD I70.2 (peripheral arterial disease)
Secondary diagnosis: ICD E10-E14 (Diabetes mellitus)
**OPS Codes**
Diagnostic Angiogram: 3-605, 3-607, 3-608
Amputation above the ankle: OPS 5-864.1, 5-864.2, 5-864.3, 5-864.4, 5-864.5, 5-864.6, 5-864.7, 5-864.8, 5-864.9, 5-864.A, 5-864.X, 5-864.Y, 5-865.0, 5-865.2, 5-865.3
Amputation below the ankle: OPS 5-865.1, 5-865.4, 5-865.5, 5-865.6, 5-865.8, 5-865.9, 5-865.7, 5-865.X, 5-865.Y
Surgical revascularization: OPS 5-381, 5-393.3, 5-393.4, 5-393.5, 5-393.6, 5-380.7, 5-380.a5
Interventional revascularization: OPS 5-38A.7, 5-38A.8, 5-38A.C, 5-38A.4, 8-836.0, 8-836.3, 8-836.7, 8-836.8, 8-836.P, 8-84+any following, 8-836.1, 8-836.2, 8-83B.4, 8-83B.9, 8-83B.BA, 8-83B.BD, 8-83B.BB, 8-83B.BC, 8-83B.F1, 8-83B.F2, 8-83B.08, 8-83B.4, 8-83B.4, 8-83B.A1, 8-83B.B6, 8-83B.06, 8-83B.E0, 8-83B.71, 8-83B.EX, 8-83B.BX, 8-83c, 8-83B.D, 8-83B.50, 8-83B.52, 8-83B.0B
Revision surgery: OPS 5-866.3, 5-866.4, 5-866.5, 5-866.X, 5-866.Y, 5-894, 5-895, 5-896, 5-921, 5-394, 8-192, 5-388.7, 5-388.8, 5-380.5, 5-380.8, 5-380.a6, 5-380.a7, 5-850, 5-869.1, 5-852, 5-780.6, 5-892.0, 5-892.x

Table S1: **Identification of main and relevant secondary diagnoses and Procedure codes.** "OPS" for "Operationen und Prozedurenschlüssel", a German procedural coding system.

	Overall cohort	Missing allocation	Supra-inguinal lesion	Sub-inguinal lesion	Infra-popliteal lesion	Lesion at two levels	Lesion at all levels	P value
**Total no. of patients**	2,067	251 (12.1)	133 (6.4)	275 (13.3)	258 (12.5)	870 (42.1)	280 (13.6)	
**Age** n (%), **overall***	68 (61-78)	70 (60-78)	59 (55-65)	67 (61-73)	75 (66-82)	70 (62-79)	67 (60.5-74)	<0.001 #
≤ 59	404 (19.6)	62 (24.7)	69 (51.9)	52 (18.9)	27 (10.5)	135 (15.5)	59 (21.1)	<0.001
60–74	961 (46.5)	97 (38.7)	54 (40.6)	160 (58.2)	100 (38.8)	392 (45.1)	158 (56.4)	<0.001
≥ 75	702 (34.0)	92 (36.7)	10 (7.5)	63 (22.9)	131 (50.8)	343 (39.4)	63 (22.5)	<0.001
**Body mass index** kg/m² *	26 (23-30)	24 (20-31)	22 (21-30)	29 (23-38)	28.5 (26-32)	26 (23-29)	26 (22-29)	0.109
**Frailty at first admission** n (%) 1	1,518 (73.4)	128 (51.0)	120 (90.2)	243 (88.4)	161 (62.4)	654 (75.2)	212 (75.7)	<0.001
2	433 (21.0)	89 (33.5)	10 (7.5)	29 (10.6)	74 (28.7)	176 (20.2)	55 (19.6)	<0.001
3	116 (5.6)	34 (13.6)	3 (2.3)	3 (1.1)	23 (8.9)	40 (4.6)	13 (4.6)	<0.001
**Coronary heart disease** n (%)	753 (36.4)	79 (31.5)	27 (20.3)	91 (33.1)	91 (35.3)	334 (38.4)	131 (46.8)	<0.001
**Diabetes mellitus** n (%)	941 (45.5)	93 (37.1)	30 (22.6)	116 (42.2)	144 (55.8)	419 (48.2)	139 (49.6)	<0.001
**Arterial hypertonus** n (%)	1,610 (77.9)	148 (59.0)	107 (80.5)	225 (81.8)	189 (73.3)	700 (80.5)	241 (86.1)	<0.001
**Chronic kidney disease** n (%)	951 (46.0)	81 (32.3)	29 (21.8)	113 (41.1)	149 (57.8)	447 (51.4)	132 (47.1)	<0.001
**Hypercholesterolemia** n (%)	1,555 (75.2)	121 (48.2)	100 (75.2)	235 (85.5)	167 (64.7)	692 (79.5)	240 (85.7)	<0.001
**Medication; n with information on medication**	841	10	38	95	114	409	175	
**Antiplatelet** any n(%)	459 (54.6)	3 (30.0)	13 (34.2)	29 (30.5)	56 (49.1)	235 (57.5)	123 (70.3)	<0.001
**Antiplatelet** mono n(%)	313 (37.2)	3 (30.0)	11 (29.0)	20 (21.1)	24 (21.1)	166 (40.6)	89 (50.9)	<0.001
**DOAK or Vit.K-antagonist** n(%)	71 (8.4)	0	1-3 (2.6)	3 (3.2)	17 (14.9)	37 (9.1)	13 (7.4)	0.027
**Statin** n(%)	595 (70.8)	6 (60.0)	31 (81.6)	81 (85.3)	79 (69.3)	280 (68.5)	118 (67.4)	0.013
**No. of females** n (%)	595 (28.8)	72 (28.7)	49 (36.8)	84 (30.6)	78 (30.2)	255 (29.3)	57 (20.4)	0.012

**PAD stage at first admission (Fontaine)** n (%) IIa	94 (4.6)	8 (3.3)	8 (6.0)	21 (7.6)	14 (5.4)	31 (3.6)	12 (4.3)	<0.001
IIb	926 (44.9)	64 (26.0)	105 (79.0)	193 (70.2)	57 (22.1)	393 (45.2)	113 (40.4)	<0.001
III	270 (13.1)	25 (10.2)	11 (8.3)	18 (6.6)	21 (8.1)	137 (15.8)	56 (20.0)	<0.001
IV	729 (35.4)	109 (44.3)	9 (6.8)	42-4 (15.3)	166 (64.3)	307-309 (35.3)	99 (35.4)	<0.001
unknown	42 (2.0)	39 (15.9)	0	1-3 (0.4)	0	2-4 (0.2)	0	<0.001
**No of admissions** n (%), 1	1,411 (68.3)	230 (91.6)	116 (87.2)	211 (76.7)	196 (76.0)	532 (61.2)	126 (45.0)	<0.001
2	400 (19.4)	17 (6.8)	13 (9.8)	48 (17.5)	52 (20.2)	201 (23.1)	69 (24.6)	<0.001
≥ 3	258 (12.5)	4 (1.6)	4 (3.0)	16 (5.8)	10 (3.9)	137 (15.7)	43 (15.4)	<0.001
**Primary revascularization** n (%), Interventional	253 (12.2)	13 (5.2)	6 (4.5)	35 (12.7)	24-6 (9.3)	133 (15.3)	42 (15.0)	<0.001
Surgical	215 (10.4)	108 (43.0	1-3 (0.8)	14-16 (5.1)	1-3 (0.4)	52 (6.0)	39 (13.9)	<0.001
Both interventional + surgical	97 (4.7)	36 (14.3)	2-4 (1.5)	2-4 (0.7)	0	33 (3.8)	24 (8.6)	<0.001

Table S2: Patient level: Patient characteristics by primary anatomical location of arterial atherosclerotic lesion. *values are median (interquartile range). P values are results from chi squared test for categorial strata or exact tests, where appropriate, and are non-parametric tests for continuous variables (#). Both interventional + surgical in maximum revascularization refers to overall performance of both types of revascularization, not necessarily during one admission. Numbers smaller than 3 were changed into a range. The percentage was randomly chosen as that of one of the numbers within the range. Available data for medication was N = 841, for BMI N=286. In case of medication, the total (100%) is considered 841.

	**Overall cohort**	**Missing allo-cation**	**Supra-inguinal lesion**	**Sub-inguinal lesion**	**Infra-popliteal lesion**	**Lesion at two levels**	**Lesion at all levels**
**No. of patients**	2,067	251 (12.1)	133 (6.4)	275 (13.3)	258 (12.5)	870 (42.1)	280 (13.6)
**In-hospital death** n (%)	97 (4.7)	35 (13.9)	3-5 (2.3)	2-4 (0.7)	15 (5.8)	34 (3.9)	8 (2.9)
**Death in follow-up** n (%)	536 (27.2 of 1,970)	56 (25.9)	7 (5.4)	48 (17.6)	85 (35.0)	257 (30.7)	83 (30.5)
.. among those with major limb event n (%) of n limb events #	52 (37.4) of 139	10 (50.0) of 20	0 of 1-2	3 (30.0) of 10	2-3 (28.6) of 7	18 (34.6) of 52	17 (34.7) of 49

.. among those with minor limb event n (%) of n limb events #	109 (42.9) of 254	12 (21.4) of 56	0 of 5	2-3 (22.2) of 9	25 (55.6) of 45	52 (52.5) of 99	18 (45.0) of 40
**Endpoints by primary Revascularization**							
**None** (N)	1,502 (72.7)	94 (37.5)	124 (93.2)	224 (81.5)	233 (90.3)	652 (74.9)	175 (62.5)
Amputation-free survival	1,025 (68.2)	43 (45.7)	115 (92.7)	184 (82.1)	138 (59.2)	428 (65.6)	117 (66.9)
Time to major amputation or death (days) *	323 (67-768)	84.5 (7-367)	255 (39-814)	484.5 (177.5-1,025.5)	275.5 (43-702)	347 (91-864.5)	445.5 (38-721)
Overall survival	1,043 (69.4)	46 (48.9)	115 (92.7)	185 (82.6)	143 (61.4)	432 (66.3)	122 (69.7)
Time to death (days) *	374 (96-816)	144 (35-546.5)	255 (39-814)	524 (182-1,031)	345 (72-744)	403 (109.5-879.5)	469 (169-768)
**Interventional** (N)	253 (12.2)	13 (5.2)	6-7 (4.5)	35 (12.7)	24-25 (9.3)	133 (15.3)	42 (15.0)
Amputation-free survival	182 (71.9)	11 (84.6)	5 (83.3)	30 (85.7)	15 (62.5)	93 (69.9)	28 (66.7)
Time to major amputation or death (days) *	737 (299-1,205)	278 (278-278)	29	979 (299-1,016)	441 (172-581)	967 (330-1,506)	848 (303-1,169)
Overall survival	187 (73.9)	12 (92.3)	5 (83.3)	30 (85.7)	15 (62.5)	95 (71.4)	30 (71.4)
Time to death (days) *	848 (299-1,269)	278	34	979 (299-1,016)	441 (172-581)	984.5 (330-1,506)	906.5 (455-1,187)
**Surgical** (N)	215 (10.4)	108 (43.0	1-2 (0.8)	14 (5.1)	0-1 (0.4)	52 (6.0)	39 (13.9)
Amputation-free survival	136 (63.3)	70 (64.8)	0-1 (100)	9 (64.3)	0	35 (67.3)	21 (53.9)
Time to major amputation or death (days) *	205.5 (22.5-836.5)	33.5 (7.5-739)	856	1,028 (821-1,313)	38	429 (133-657)	519 (89-802)
Overall survival	147 (68.4)	76 (70.4)	0-1 (100)	9 (64.3)	0	35 (67.3)	26 (66.7)
Time to death (days) *	311.5 (34.5-977)	39 (12.5-852.5)	x	1,118 (821-1,313)	38	429 (147-1,143)	519 (104-830)
**Both interventional + surgical** (N)	97 (4.7)	36 (14.3)	2-3 (1.5)	1-2 (0.7)	0	33 (3.8)	24 (8.6)
Amputation-free survival	55 (56.7)	26 (72.2)	1-2 (100)	1-2 (50.0)	x	16 (48.5)	9-10 (41.7)
Time to major amputation or death (days) *	173 (26-602)	140.5 (4-497)	1,556.5 (1,014-2,099)	1,785	x	145 (28-602)	173 (88-407)

Overall survival	57 (58.8)	26 (72.2)	1-2 (100)	0-1 (50.0)	x	17 (51.5)	10-11 (45.8)
Time to death (days) *	249.5 (26.5-935)	140.5 (4-497)	x	1,785	x	249.5 (31.5-792.5)	196 (86-966)

Table S3: Patient level: Primary and secondary endpoints by primary anatomical location of arterial atherosclerotic lesion and by primary revascularization. All numbers are n (%). *values are median (interquartile range). # according to events on limb-level on either side of the lower extremity. Actual major and minor limb-events on patient level were higher because of lacking information on side coding of procedures. The stated level of lesions refers to either side of the lower limbs. Since for some revascularization procedures, there was no side coding, primary revascularizations by primary anatomical location of arterial atherosclerotic lesion do not add up to the total of the respective revascularizations. Time under observation refers to patient status of the primary endpoint, i.e. time under observation or time to death or time to amputation above the ankle. The analysis was based on 2,252 records with complete data on all variables. Numbers smaller than 3 were changed into a range. The percentage was randomly chosen as that of one of the numbers within the range.

	**Univariable HR for major amputation or death**		**Multivariable Cox regression for major amputation or death**	
	Hazard Ratio [95% CI]	P-value	Hazard Ratio [95% CI]	P-value
**Age** (incremental increase in years)	1.06 [1.05-1.06]	<0.001	1.05 [1.04-1.06]	<0.001
**Sex** (female as reference)	1		1	
male	0.99 [0.83-1.19]	.932	1.25 [1.03-1.51]	0.023
**Frailty score (1 as reference)**	1		1	
2	3.60 [3.01-4.32]	<0.001	1.02 [1.01-1.03]	<0.001
3	9.39 [7.20-12.26]	<0.001	1.02 [1.00-1.04]	0.010
**Primary revascularization**				
None as reference	1		1	
Interventional	0.71 [0.55-0.92]	.009	0.72 [0.55-0.94]	0.015
Surgical	1.19 [0.86-1.65]	.293	1.32 [0.95-1.83]	0.099
Both interventional and surgical	1.79 [1.24-2.58]	.002	2.24 [1.54-3.26]	<0.001
**Primary lesion**				
Infrapopliteal as reference	1		1	
Infrainguinal-to-popliteal	0.37 [0.27-0.52]	<0.001	0.62 [0.44-0.87]	0.007
Suprainguinal	0.16 [0.08-0.30]	<0.001	0.42 [0.21-0.79]	0.008
2 of 3	0.75 [0.60-0.94]	.014	0.99 [0.79-1.25]	0.950
3 of 3	0.84 [0.64-1.11]	.216	1.19 [0.88-1.59]	0.254
**PAD stage at admission**				
CLTI (Fontaine III and IV) as reference	1		1	
Claudication (Fontaine IIa and IIb)	0.26 [0.22-0.32]	<0.001	0.99 [0.98-0.99]	0.001
**Diabetes mellitus** (none as reference)	1		1	
Existing Diabetes mellitus	1.80 [1.52-2.13]	<0.001	1.02 [1.01-1.02]	<0.001

Table S4: Patient level: Hazard Ratio for Primary endpoint: Major amputation or death. Infrapopliteal location was chosen as reference due to lowest result in descriptive analysis of the primary endpoint at patient level. For the primary endpoint at patient level, there were interactions between time and diabetes mellitus, time and frailty, and time and PAD stage at admission, which were accounted for in the Cox regression using interaction terms. The analysis was based on 1,808 records with complete data on all variables. CLTI for chronic limb-threatening ischemia, surrogate parameter PAD stage Fontaine III or IV at first admission.

	**Univariable HR for death**		**Multivariable Cox regression for death**	
	Hazard Ratio [95% CI]	P-value	Hazard Ratio [95% CI]	P-value
**Age** (incremental increase in years)	1.06 [1.05-1.07]	<0.001	1.06 [1.05-1.07]	<0.001
**Sex** (female as reference)	1		1	
male	1.01 [0.84-1.22]	.901	1.35 [1.11-1.65]	0.002
**Frailty score** (1 as reference)	1		1	
2	3.25 [2.70-3.92]	<0.001	1.02 [1.01-1.02]	<0.001
3	8.19 [6.25-10.73]	<0.001	1.02 [1.01-1.04]	0.003
**Primary revascularization**				
None as reference	1		1	
Interventional	0.69 [0.53-0.90]	.006	0.74 [0.56-0.96]	0.025
Surgical	1.06 [0.75-1.49]	.743	1.20 [0.85-1.70]	0.299
Both interventional and surgical	1.66 [1.15-2.41]	.007	2.07 [1.41-3.06]	<0.001
**Primary lesion**				
Infrapopliteal as reference	1		1	
Infrainguinal-to-popliteal	0.38 [0.27-0.54]	<0.001	0.69 [0.48-0.98]	0.038
Suprainguinal	0.17 [0.09-0.32]	<0.001	0.49 [0.25-0.96]	0.038
2 of 3	0.78 [0.62-0.98]	.030	1.08 [0.85-1.36]	0.541
3 of 3	0.75 [0.57-0.99]	.048	1.13 [0.84-1.54]	0.420
**PAD stage at admission**				
CLTI (Fontaine III and IV) as reference	1		1	
Claudication (Fontaine IIa and IIb)	0.27 [0.23-0.33]	<0.001	0.99 [0.98-0.99]	<0.001
**Diabetes mellitus** (none as reference)	1		1	
Existing Diabetes mellitus	1.80 [1.51-2.12]	<0.001	1.01 [1.01-1.02]	<0.001

Table S5: Patient level: Hazard Ratio for Secondary endpoint: Death. Infrapopliteal location was chosen as reference due to lowest result in descriptive analysis of the secondary endpoint at patient level. For the secondary endpoint at patient level, there were interactions between time and diabetes mellitus and time and frailty, which were accounted for in the Cox regression using interaction terms. The analysis was based on 1,808 records with complete data on all variables. CLTI for chronic limb-threatening ischemia, surrogate parameter PAD stage Fontaine III or IV at first admission.

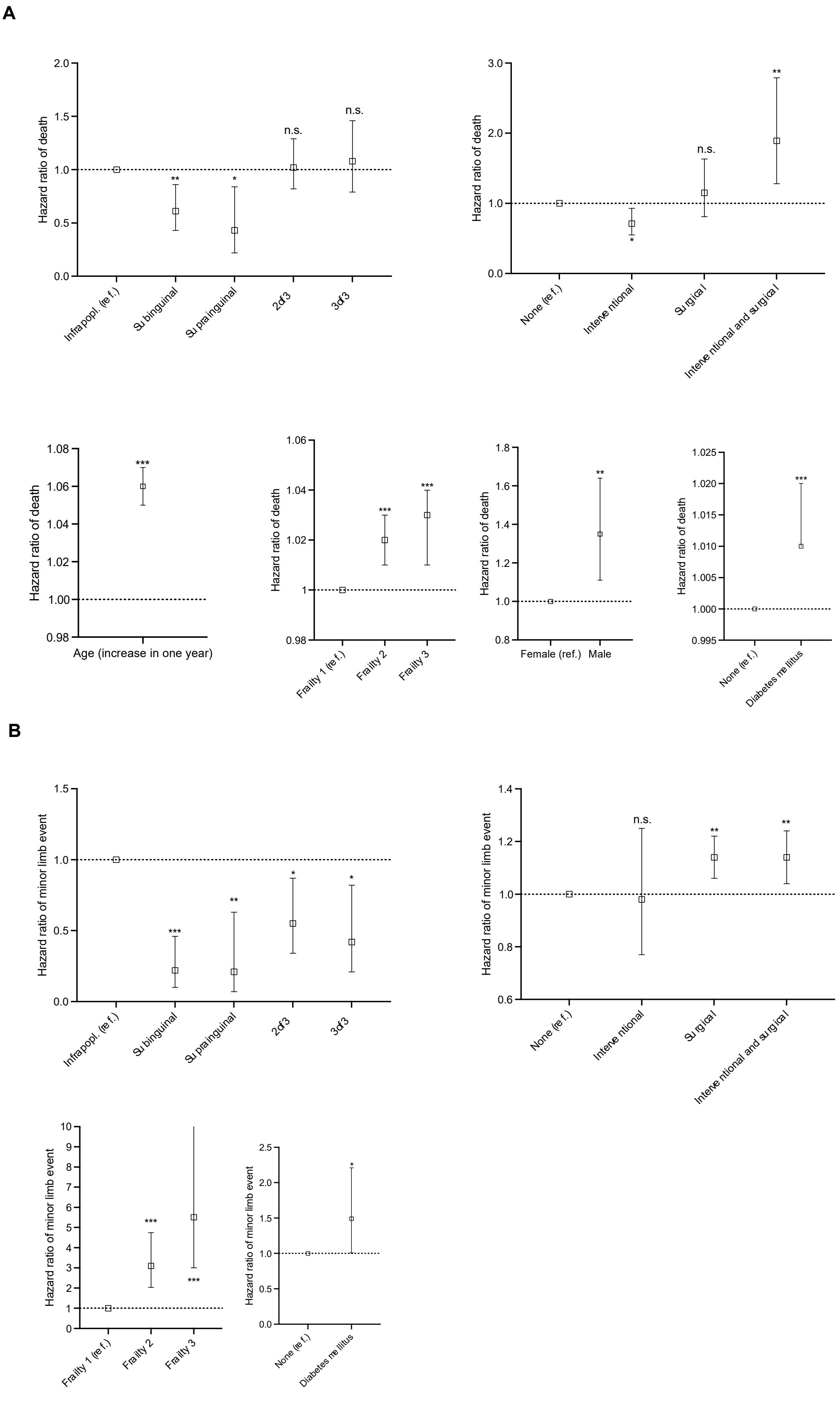
A
Hazard ratio of death
n.s.
Infrapopl. (ref.)
Subinguinal
Suprainguinal
2of3
3of3
None (ref.)
Intenentional
Surgical
Intenentional and surgical
Age (increase in one year)
Frailty 1 (ref.)
Frailty 2
Frailty 3
Female (ref.)
Male
Diabetes mellitus
B
Hazard ratio of minor limb event

Figure S2: Results from a multivariable Cox regression: Secondary endpoints. A Secondary endpoint at patient level: Overall survival. **B** Secondary endpoint at limb level: Occurrence of minor limb event. Univariable and multivariable Hazard Ratios for **A** in Supp. Table 5, univariable and multivariable Hazard Ratios for **B** in Supp. Table 9. Primary endpoints at patient and limb level in Figure 3, corresponding univariable and multivariable Hazard Ratios in Supp. Table 5 and Supp. Table 8, respectively. Ref. for reference. Infrapopl. for infrapopliteal. N.s. for not significant. The dashed lines represent Hazard Ratio = 1. * for p<0.05, ** for p<0.01, and *** for p<0.001.

	Multivariable Cox regression for major amputation or death: claudication		Multivariable Cox regression for major amputation or death: CLTI	
	Hazard Ratio [95% CI]	P-value	Hazard Ratio [95% CI]	P-value
**Age** (incremental increase in years)	1.04 [1.02-1.06]	<0.001	1.05 [1.03-1.06]	<0.001
**Sex** (female as reference)	1		1	
male	1.57 [1.05-2.34]	.029	1.15 [0.93-1.44]	0.204
**Frailty score** (1 as reference)	1		1	
2	1.01 [0.99-1.02]	.239	1.02 [1.01-1.03]	<0.001
3	1.08 [1.03-1.12]	.001	1.02 [1.00-1.04]	0.022
**Primary revascularization**				
None as reference	1		1	
Interventional	0.92 [0.60-1.41]	.696	0.68 [0.48-0.96]	0.030
Surgical	1.37 [0.77-2.43]	.281	1.28 [0.86-1.93]	0.228
Both interventional and surgical	1.75 [0.76-4.05]	.192	2.08 [1.36-3.19]	0.001
**Primary lesion**				
Infrapopliteal as reference	1		1	
Infrainguinal-to-popliteal	0.78 [0.40-1.54]	.476	0.82 [0.51-1.32]	0.405
Suprainguinal	0.30 [0.10-0.96]	.042	1.13 [0.49-2.63]	0.771
2 of 3	0.94 [0.51-1.76]	.857	1.11 [0.86-1.43]	0.421
3 of 3	0.97 [0.48-1.98]	.943	1.40 [1.00-1.94]	0.047
**Diabetes mellitus** (none as reference)	1		1	
Existing Diabetes mellitus	1.02 [1.00-1.03]	.004	1.01 [1.00-1.02]	0.004
	**Multivariable Cox regression for death: claudication**		**Multivariable Cox regression for death: CLTI**	
	Hazard Ratio [95% CI]	P-value	Hazard Ratio [95% CI]	P-value
**Age** (incremental increase in years)	1.05 [1.03-1.07]	<0.001	1.05 [1.04-1.06]	<0.001
**Sex** (female as reference)	1		1	
male	1.61 [1.07-2.42]	.023	1.29 [1.03-1.62]	0.027
**Frailty score** (1 as reference)	1		1	
2	1.01 [0.99-1.02]	.242	1.02 [1.01-1.03]	<0.001
3	1.08 [1.03-1.12]	.001	1.02 [1.01-1.04]	0.008
**Primary revascularization**				
None as reference	1		1	
Interventional	0.87 [0.56-1.35]	.539	0.73 [0.51-1.03]	0.073
Surgical	1.33 [0.74-2.41]	.343	1.12 [0.73-1.73]	0.596
Both interventional and surgical	1.39 [0.55-3.48]	.484	2.00 [1.29-3.10]	0.002
**Primary lesion**				
Infrapopliteal as reference	1		1	
Infrainguinal-to-popliteal	0.80 [0.41-1.57]	.513	0.88 [0.54-1.45]	0.624
Suprainguinal	0.32 [0.10-1.02]	.054	1.43 [0.62-3.33]	0.404
2 of 3	0.96 [0.51-1.78]	.887	1.20 [0.93-1.56]	0.155

3 of 3	0.77 [0.37-1.61]	.481	1.37 [0.97-1.92]	0.073
**Diabetes mellitus** (none as reference)	1		1	
Existing Diabetes mellitus	1.01 [1.00-1.03]	.006	1.01 [1.00-1.02]	0.006

Table S6: Patient level: Hazard Ratio for Primary endpoint, stratified by PAD stage at admission: Major amputation or death. Stratification was by claudication (Fontainte Stage IIa and IIb) and by chronic limb-threatening ischemia, CLTI (Fontaine stage III and IV). Infrapopliteal location was chosen as reference due to lowest result in descriptive analysis of the primary endpoint at patient level. For the primary endpoint at patient level, there were interactions between time and diabetes mellitus and time and frailty, which were accounted for in the Cox regression using interaction terms, which were maintained in the stratification. The analysis was based on 1,808 records with complete data on all variables, of which 948 were claudication and 860 were CLTI; unknown PAD stages were excluded from this analysis (overall unknown PAD stage n=48, Table 1). Addition of BMI or medication to the model in subgroups with information on these parameters did not increase accuracy in a likelihood ratio test.

	**Overall cohort**	**Missing allo-cation**	**Supra-inguinal lesion**	**Sub-inguinal lesion**	**Infra-popliteal lesion**	**Lesion at two levels**	**Lesion at all levels**
**No. of limbs**	2,633	380 (14.4⇐)	304 (11.6⇐)	482 (18.3⇐)	299 (11.4⇐)	949 (36.0⇐)	219 (8.3⇐)
**Endpoints by primary Revascularization**							
**None**	2,189 (83.1)	134 (35.3)	290 (95.4)	450 (93.4)	291 (97.3)	851 (89.7)	173 (79.0)
Major adverse limb event	45 (2.1)	18 (13.4)	0-1 (0.3)	0-1 (0.2)	7 (2.4)	11 (1.3)	7 (4.1)
Time to major adverse limb event (days) *	16 (6-36)	8 (5-21)	x/1	x/6	23 (16-41)	22 (6-67)	19 (5-138)
Minor limb event	190 (8.7)	43 (32.1)	9 (3.1)	4 (0.9)	48 (16.5)	73 (8.6)	13 (7.5)
Time to minor limb event (days) *	4 (0-14)	1 (0-26)	0 (0-4)	4 (0-10)	4.5 (0-8.5)	6 (0-17)	0 (0-16)
**Interventional**	127 (4.8)	66 (17.4)	8 (2.6)	16 (3.3)	7 (2.3)	22 (2.3)	8 (3.7)
Major adverse limb event	35 (27.6)	15 (22.7)	3-4 (37.5)	5 (31.3)	0-1 (14.3)	8 (36.4)	3 (37.5)
Time to major adverse limb event (days) *	290 (161-406)	337 (153-387)	298 (137-769)	406 (364-513)	406	222.5 (68-431.5)	265 (216-285)
Minor limb event	11 (8.7)	4 (6.1)	0	0	3 (42.9)	1-2 (9.1)	1-2 (25.0)
Time to minor limb event (days) *	0 (0-14)	0 (0-59.5)	x	x	4 (0-5)	7 (0-14)	108 (0-216)
**Surgical**	233 (8.9)	137 (36.1)	6 (2.0)	12 (2.5)	0-1 (0.3)	50 (5.3)	27 (12.3)
Major adverse limb event	65 (27.9)	19 (13.9)	4 (66.7)	7 (58.3)	0	20 (40.0)	15 (55.6)

Time to major adverse limb event (days) *	161 (65-384)	81 (53-260)	364.5 (224.5-639)	252 (65-896)	x	163 (58-327.5)	310 (106-516)
Minor limb event	87 (37.3)	51 (37.2)	0	5 (41.7)	0	23 (46.0)	8 (29.6)
Time to minor limb event (days) *	0 (0-2)	0 (0-0)	x	6 (0-554)	x	0 (0-57)	0 (0-13.5)
**Both interventional + surgical**	84 (3.2)	43 (11.3)	0	4 (0.8)	0	26 (2.7)	11 (5.0)
Major adverse limb event	19 (22.6)	3 (7.0)	0	4 (100.0)	0	9 (34.6)	3 (27.3)
Time to major adverse limb event (days) *	151 (54-435)	435 (224-871)	x	280.5 (26-576.5)	x	141 (54-197)	61 (61-652)
Minor limb event	32 (38.1)	13 (30.2)	0	4 (100.0)	0	8 (30.8)	7 (63.6)
Time to minor limb event (days) *	0 (0-33)	0 (0-0)	x	155 (27.5-353)	x	0 (0-14)	5 (0-83)

Table S7: Limb level: Primary and secondary endpoints by primary anatomical location of arterial atherosclerotic lesion and by primary revascularization. All numbers are n (%). *values are median (interquartile range). Since for some revascularization procedures, there was no side coding, primary revascularizations by primary anatomical location of arterial atherosclerotic lesion do not add up to the total of the respective revascularizations. Time under observation refers to limb status of the primary endpoint, i.e. time under observation or time to major adverse limb event. Numbers smaller than 3 were changed into a range. The percentage was randomly chosen as that of one of the numbers within the range.

	**Univariable HR for major adverse limb event**		**Multivariable Cox regression for major adverse limb event**	
	Hazard Ratio [95% CI]	P-value	Hazard Ratio [95% CI]	P-value
**Frailty score** (1 as reference)	1		1	
2	2.72 [1.77-4.16]	<0.001	0.99 [0.94-1.04]	0.605
3	7.70 [3.53-13.88]	<0.001	1.14 [1.02-1.28]	0.017
**Primary revascularization**				
None as reference	1		1	
Interventional	14.7 [8.5-15.6]	<0.001	2.16 [1.70-2.74]	<0.001
Surgical	20.97 [13.2-33.3]	<0.001	2.15 [1.69-2.72]	<0.001
Both interventional and surgical	18.97 [9.8836.41]	<0.001	2.12 [1.66-2.70]	<0.001
**Primary lesion**				
3 of 3 as reference	1		1	
Infrapopliteal	0.39 [0.18-0.86]	.019	0.85 [0.37-1.96]	0.699
Infrainguinal-to-popliteal	0.41 [0.23-0.76]	.004	0.51 [0.27-0.98]	0.044
Suprainguinal	0.57 [0.36-0.91]	.018	0.54 [0.24-1.21]	0.134
2 of 3	0.40 [0.18-0.89]	.024	0.92 [0.55-1.51]	0.730

Table S8: Limb level: Hazard Ratio for Primary endpoint: Major adverse limb event. 3 of 3 was chosen as reference due to highest result in descriptive analysis of the primary endpoint at limb level.

For the primary endpoint at limb level, there were interactions between time and primary revascularization and time and frailty, which were accounted for in the Cox regression using an interaction term. The analysis was based on 2,252 records with complete data on all variables.

	**Univariable HR for minor limb event**		**Multivariable Cox regression for minor limb event**	
	Hazard Ratio [95% CI]	P-value	Hazard Ratio [95% CI]	P-value
**Age** (incremental increase in years)	0.97 [0.95-0.98]	<0.001	0.99 [0.97-1.01]	0.282
**Frailty score** (1 as reference)	1		1	
2	3.78 [2.51-5.71]	<0.001	3.10 [2.03-4.74]	<0.001
3	7.31 [4.09-13.06]	<0.001	5.51 [3.00-10.09]	<0.001
**Primary revascularization**				
None as reference	1		1	
Interventional	1.05 [0.38-2.86]	.926	0.98 [0.77-1.25]	0.855
Surgical	2.00 [1.13-3.52]	.017	1.14 [1.06-1.22]	0.001
Both interventional and surgical	4.69 [2.43-9.05]	<0.001	1.14 [1.04-1.24]	0.007
**Primary lesion**				
Infrapopliteal as reference	1		1	
Infrainguinal-to-popliteal	0.17 [0.08-0.35]	<0.001	0.22 [0.10-0.46]	<0.001
Suprainguinal	0.16 [0.05-0.44]	.001	0.21 [0.07-0.63]	0.005
2 of 3	0.49 [0.31-0.78]	.003	0.55 [0.34-0.87]	0.010
3 of 3	0.37 [0.19-0.72]	.003	0.42 [0.21-0.82]	0.012
**Diabetes mellitus** (none as reference)	1		1	
Existing Diabetes mellitus	1.65 [1.12-2.43]	.011	1.49 [1.01-2.21]	0.045

Table S9: Limb level: Hazard Ratio for Secondary endpoint: Minor limb event. Infrapopliteal location was chosen as reference due to highest result in descriptive analysis of the secondary endpoint at limb level. For the secondary endpoint at limb level, there was an interaction between time and revascularization, which was accounted for in the Cox regression using an interaction term. The analysis was based on 2,154 records with complete data on all variables.
